# Transfer learning for clustering single-cell RNA-seq data crossing-species and batch, case on uterine fibroids

**DOI:** 10.1093/bib/bbad426

**Published:** 2023-11-22

**Authors:** Yu Mei Wang, Yuzhi Sun, Beiying Wang, Zhiping Wu, Xiao Ying He, Yuansong Zhao

**Affiliations:** Department of Gynecology, Shanghai First Maternity and Infant Hospital, School of Medicine, Tong Ji University, Shanghai , China; Shanghai Key Laboratory of Maternal and Fetal Medicine, Shanghai First Maternity and Infant Hospital, Shanghai,China; School of Computer Science and Technology, Harbin Institute of Technology, Harbin, China; Department of Gynecology, Shanghai First Maternity and Infant Hospital, School of Medicine, Tong Ji University, Shanghai , China; Shanghai Key Laboratory of Maternal and Fetal Medicine, Shanghai First Maternity and Infant Hospital, Shanghai,China; Department of Gynecology, Shanghai First Maternity and Infant Hospital, School of Medicine, Tong Ji University, Shanghai , China; Shanghai Key Laboratory of Maternal and Fetal Medicine, Shanghai First Maternity and Infant Hospital, Shanghai,China; Department of Gynecology, Shanghai First Maternity and Infant Hospital, School of Medicine, Tong Ji University, Shanghai , China; Shanghai Key Laboratory of Maternal and Fetal Medicine, Shanghai First Maternity and Infant Hospital, Shanghai,China; University of Texas Health Science Center at Houston, 77030-5400, USA

**Keywords:** single-cell RNA-seq data, uterine fibroids, transfer learning, batch effect, graph convolutional networks

## Abstract

Due to the high dimensionality and sparsity of the gene expression matrix in single-cell RNA-sequencing (scRNA-seq) data, coupled with significant noise generated by shallow sequencing, it poses a great challenge for cell clustering methods. While numerous computational methods have been proposed, the majority of existing approaches center on processing the target dataset itself. This approach disregards the wealth of knowledge present within other species and batches of scRNA-seq data. In light of this, our paper proposes a novel method named graph-based deep embedding clustering (GDEC) that leverages transfer learning across species and batches. GDEC integrates graph convolutional networks, effectively overcoming the challenges posed by sparse gene expression matrices. Additionally, the incorporation of DEC in GDEC enables the partitioning of cell clusters within a lower-dimensional space, thereby mitigating the adverse effects of noise on clustering outcomes. GDEC constructs a model based on existing scRNA-seq datasets and then applying transfer learning techniques to fine-tune the model using a limited amount of prior knowledge gleaned from the target dataset. This empowers GDEC to adeptly cluster scRNA-seq data cross different species and batches. Through cross-species and cross-batch clustering experiments, we conducted a comparative analysis between GDEC and conventional packages. Furthermore, we implemented GDEC on the scRNA-seq data of uterine fibroids. Compared results obtained from the Seurat package, GDEC unveiled a novel cell type (epithelial cells) and identified a notable number of new pathways among various cell types, thus underscoring the enhanced analytical capabilities of GDEC.

Availability and implementation: https://github.com/YuzhiSun/GDEC/tree/main

## INTRODUCTION

Traditional bulk RNA sequencing measures the average gene expression levels of all cells in a sample, which can mask the diversity and functional heterogeneity of individual cells [1]. Single-cell RNA sequencing (scRNA-seq) is a powerful and revolutionary technique that allows researchers to study the transcriptomes of individual cells within a heterogeneous population [2]. scRNA-seq enables the identification of distinct cell types, subtypes and even rare cell populations that might be overlooked in bulk analysis. It also provides a comprehensive catalog of cell states and lineage trajectories, crucial for understanding tissue and organ organization and their functional implications [3]. Moreover, scRNA-seq helps unravel complex biological processes by analyzing gene expression profiles at the single-cell level during organism or tissue development [4]. This approach reveals insights into lineage relationships and differentiation trajectories of different cell types. Additionally, scRNA-seq has immense potential in uncovering cellular and molecular mechanisms underlying diseases such as cancer, neurodegenerative disorders and autoimmune diseases [5]. It can identify disease-specific cell types, biomarkers and potential therapeutic targets. Furthermore, single-cell profiling identifies unique cellular features that could be targeted for therapeutic intervention, offering promise for personalized treatment strategies based on individual-specific responses to drugs [6, 7]. In summary, scRNA-seq significantly advances our understanding of cellular heterogeneity and finds crucial applications in various fields of biology and medicine. It has become an important means to study organ development and disease mechanisms [8].

However, the identification of cell types in scRNA-seq data has become challenging due to limitations posed by the sparse and high-dimensional gene expression matrix, as well as the high noise caused by low-depth sequencing [9]. To address this issue, researchers have developed numerous computational methods for cell clustering of scRNA-seq data. These methods can be broadly categorized into three groups.

The first group consists of traditional clustering methods, with many of them being based on the K-means clustering algorithm. For instance, Sun *et al.* [10] introduced the low-rank self-expression K-means method, which seeks a low-rank representation of the original data by using an optimization algorithm based on the enhanced Lagrangian multiplier to represent potential relationships between cells, without directly calculating the similarity between cells. Hicks *et al.* [11] proposed mbkmeans (mini-batch k-means), an open-source implementation of the small-batch k-means algorithm. This method addresses the slow or inoperable processing of large datasets, which is a common issue with standard K-means. Wang *et al.* [12] developed single-cell bisecting K-means clustering (scBKAP), which leverages an autoencoder network and the bisected K-means clustering method. This approach not only mitigates the dropout problem in scRNA-seq data but also reduces the dimensionality of the reconstructed data. Another notable method is SIMLR, introduced by Wang *et al.* [13], which is based on K-means and is applicable for dimensionality reduction, clustering and visualization. One of the widely used packages for this type of method is the Seurat package [14], which also employs K-means for the clustering of scRNA-seq data.

The second type of method is based on similarity, where these methods rely on similarity measures to determine the similarity between cells. Kim *et al.* [15] investigated the impact of five similarity metrics (Euclidean, Manhattan and maximum distances, as well as Pearson and Spearman’s correlation coefficients) on clustering results. He *et al.* [16] introduced cluster similarity profiles based on transcriptome similarity between each single cell and the average of each cell cluster. Cao *et al.* [17] proposed SAILERX, which not only performs clustering but also corrects technical noise in the sequencing process using local structural similarity theory with pairwise similarity measurement of the two modalities. Pouyan [18] developed a random forest-based method called RAFSIL, which clusters cells based on their similarity. It is worth mentioning that the traditional clustering method Single-cell Interpretation via Multi-kernel LeaRning (SIMLR) [13] is also based on kernel similarity. Overall, both of these methods require the presetting of the number of categories and often involve significant computational and time costs. Additionally, since the cellular state is constantly changing, relying solely on correlation coefficients to map the relationship between cells might not be sufficient.

The most powerful category of methods for cell clustering in scRNA-seq data is based on deep learning. These methods directly utilize autoencoders and decoders, showcasing strong robustness. Kopf *et al.* [19] introduced the Mixture of Experts Similarity Variational Autoencoder, which utilizes a variational autoencoder coupled with a decoder based on a Mixture of Experts architecture. Wang *et al.* [20] proposed scDCCA, which integrates a deionizing autoencoder and a dual contrastive learning module to achieve cell clustering in an end-to-end manner. The autoencoder is employed to extract low-dimensional features, and the double-contrastive learning module captures pairwise proximity of cells. Hu *et al.* [21] combined attribute information and structural information of cells in their approach called single-cell deep fusion clustering model (scDFC), which includes an attribute feature clustering module and a structure attention feature clustering module. Zeng *et al.* [22] presented automatic deep embedding clustering (ADClust), where a pre-trained autoencoder is utilized to obtain a low-dimensional representation of cells, and statistical tests are used to merge similar micro-clusters into larger clusters. While deep learning-based methods have achieved significant success in clustering scRNA-seq data, there is already a vast amount of scRNA-seq data from different species. Some of this data has been accurately labelled through biological experimental methods. Addressing how to leverage existing data to improve the clustering quality of new scRNA-seq data remains an urgent challenge. Additionally, it is crucial to explore how to utilize information from different batches of scRNA-seq data to enhance comparability between datasets.

To address these challenges, we have developed a novel method called, graph-based deep embedding clustering (GDEC) that aims to perform clustering of single-cell RNA-seq data across different species and batches. ‘GDEC’ combines the power of graph convolutional networks (GCN) and DEC. Firstly, we use GCN to extract the topology of the gene interaction network, which helps in capturing the relationships between genes and their interactions. Next, we employ DEC, which utilizes a deep autoencoder to learn a low-dimensional embedding of the scRNA-seq data. This low-dimensional representation helps in preserving the essential information while reducing the noise and dimensionality of the data. To fully leverage the wealth of information from previous scRNA-seq datasets and accurately labelled scRNA-seq datasets (Gold Standard Dataset), we apply transfer learning in our method. GDEC can acquire significant prior knowledge from existing datasets, enabling a better understanding of gene expression patterns and cellular relationships between different cell types. The application of transfer learning allows GDEC to achieve cross-species and cross-batch scRNA-seq data clustering effectively. This means that the method can identify and group cells from different species and experimental batches, even in scenarios where labelled data is limited, thanks to the knowledge gained from previous datasets. Ultimately, this contributes to more accurate and comprehensive clustering results in single-cell RNA-seq analysis.

## METHODS AND MATERIALS

### Workflow of GDEC

**Figure 1:**
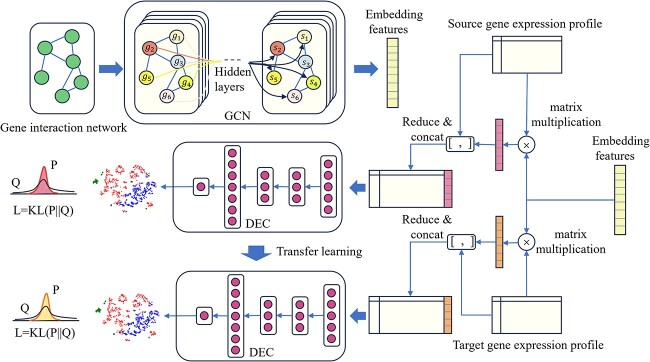
The structure of GDEC.

The workflow of GDEC is shown in Figure 1, and it consists of three main parts. Firstly, GCN is employed to extract features from the gene interaction network, yielding node-level features for each gene. Subsequently, integration takes place between the source gene expression profiles and the gene node features. The fused features are utilized to train an encoder–decoder structural model, from which the encoder architecture is extracted to facilitate dimensionality reduction of the features. The integration of the encoder with an unsupervised clustering model establishes the framework of differential expression clustering (DEC). Ultimately, the model pre-trained on the source dataset is transferred to the target dataset, where it undergoes fine-tuning and clustering, culminating in the derivation of clustering outcomes specific to the target dataset.

### Gene interaction network topology extraction by GCN

GCN has the capability to capture intricate inter-node information. Thus, we employ GCN to extract inter-gene interaction relationships. We adopt a progressive approach with two consecutive graph convolutional modules to capture the network topological characteristics of genes. The detailed formulation within each graph convolutional block is as follows:


(1) \begin{equation*} {h}_i^{\left(l+1\right)}=\sigma \left({b}^{(l)}+\sum_{j\in N(i)}\frac{1}{c_{ji}}{h}_j^{(l)}{W}^{(l)}\right) \end{equation*}

where $N(i)$ is the set of neighbors of node $i$, ${c}_{ji}$ is the product of the square root of node degrees and $\sigma$ is an activation function.

**Figure 2:**
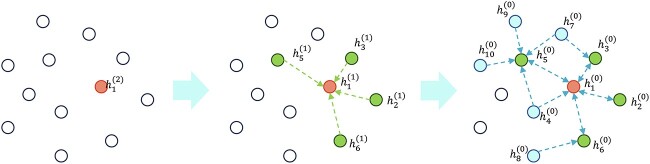
Process for constructing mini-batch graph nodes.

In consideration of the model’s robustness, particularly its ability to train on large graphs with limited computational resources; we have implemented a method of random mini-batch training for the GCN. The process is shown as Figure 2. Assuming the utilization of an L-layer graph neural network with a hidden layer size of H on a graph comprising N nodes, the storage requirement for hidden layers amounts to O(NLH). Given the potential memory overflow when N is substantial, addressing this concern becomes imperative. To tackle this issue, the development of neighbour sampling techniques is requisite in constructing mini-batch graph nodes. With each iteration of gradient descent, a random subset of graph nodes is selected, upon which computations are performed at the L-th layer of the graph neural network. Following this, in the L-1 layer of the network, the neighbouring nodes of this batch of nodes (either partial or complete) are chosen. This process is iteratively repeated until the input layer is reached.

By employing GCN, it becomes feasible to derive a feature vector for each gene. We use randomly generated features as the initial features of gene nodes. The multiplication of this vector with the sample feature vector from the gene expression profiles yields a feature vector representing the interrelation of genes within the sample. The specific formulation is outlined as follows:


(2) \begin{equation*} D=S\cdot M,S\in{\mathbb{R}}^{a\times g},M\in{\mathbb{R}}^{g\times d} \end{equation*}

where M denotes the gene feature embedding matrix obtained through GCN, S represents the gene expression profile matrix and D signifies the matrix encompassing gene interaction feature embeddings corresponding to each cell within the expression profile. Here, ‘a’ signifies the count of cells in the expression profile, ‘g’ represents the count of genes and ‘d’ signifies the dimensionality of the GCN feature embedding. Depending on distinct contextual scenarios, dimensionality reduction of D to a one-dimensional representation can be undertaken, as elucidated below:


(3) \begin{equation*} {D}^{\prime }={\sum}_{i=1}^d{D}_i,{D}^{\prime}\in{\mathbb{R}}^a \end{equation*}

Concatenating the GCN-derived features with the gene expression profile data yields the input data for the DEC model. The specific formulation is presented as follows:


(4) \begin{equation*} I=\left[S,D\right] or\ I=\left[S,{D}^{\prime}\right] \end{equation*}

### Gene expression embedding by DEC

Gene expression profile data often exhibit high-dimensional characteristics. Common unsupervised machine learning techniques such as K-means and Gaussian Mixture Models may suffer diminished effectiveness when confronted with such data. Hence, we have adopted the DEC approach to effectuate feature representation and cluster assignment on the high-dimensional gene expression profiles. DEC undertakes a mapping of the data space to a lower-dimensional feature space and iteratively optimizes clustering objectives within this feature space.

The process commences by conducting pre-training on the dataset using an encoder-decoder autoencoder, yielding a model capable of encoding and decoding data features. Subsequently, the encoder component is extracted for use as the dimensionality reduction model. The reduced feature representation is input into K-means to perform clustering, generating a set of cluster centers for initialization. Lastly, fine-tuning of the encoder model parameters is executed through the utilization of the Kullback–Leibler (KL) divergence loss function.

Employing the Student’s t-distribution as a kernel facilitates the measurement of similarity between embedded points and centroids. This choice of kernel serves to underpin the similarity assessment between the transformed embeddings and cluster centroids.


(5) \begin{equation*} {q}_{ij}=\frac{{\left(1+\parallel{z}_i-{\mu}_j{\parallel}^2/\alpha \right)}^{\frac{\alpha +1}{2}}}{\sum_{j\prime }{\left(1+\parallel{z}_i-{\mu}_{j\prime }{\parallel}^2/\alpha \right)}^{\frac{\alpha +1}{2}}} \end{equation*}

(6) \begin{equation*} {p}_{ij}=\frac{q_{ij}^2/{f}_j}{\sum_{j\prime }{q}_{ij\prime}^2/{f}_{j\prime }} \end{equation*}

(7) \begin{equation*} {f}_j={\sum}_i qij \end{equation*}

where ${z}_i$ represents the nonlinear mapping of the original features obtained through the encoder，${\mu}_j$ represents the centroids initialized using the K-means algorithm，$\alpha$represents the degrees of freedom of the Student’s t-distribution, and ${q}_{ij}$ denotes the probability of assigning sample i to cluster j (soft assignment). The loss function is defined as the KL divergence between the soft assignment ${q}_i$and the auxiliary distribution ${p}_i$:


(8) \begin{equation*} Loss= KL (P\mid | \ Q )={\sum}_i{\sum}_j{p}_{i,j}\mathit{\log}\frac{p_{ij}}{q_{ij}} \end{equation*}

### Transfer leaning for clustering scRNA-seq data across different species and batches

For a given biological organization, there exists a substantial volume of single-cell data spanning diverse species. Leveraging this wealth of data holds the potential to enhance the accuracy of cell type clustering. Consequently, the GDEC method employs transfer learning to predict data with limited sample size, facilitating cross-species cell type clustering.

Simultaneously, different library preparation strategies can lead to disparities in sequencing results for the same cell type, thereby influencing downstream analyses. In contrast to conventional techniques like canonical correlation analysis (CCA), transfer learning has the capacity to assimilate latent contextual knowledge, thereby mitigating batch effects and accomplishing tasks across diverse batches.

**Figure 3:**
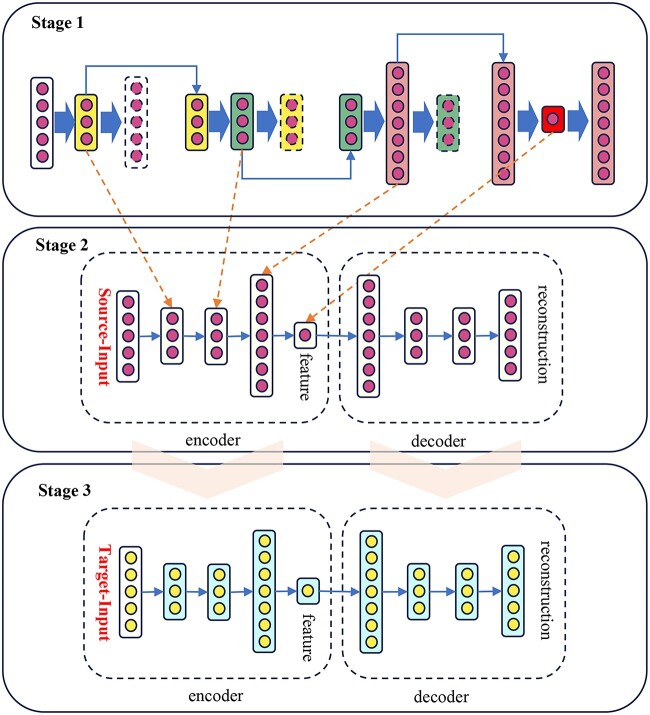
Process for transfer learning.

As illustrated in the Figure 3, our approach entails training an autoencoder model on the source data (the gold standard data). Subsequently, the architecture and parameters of this model are transferred to the target data, where fine-tuning is executed to adapt the model to the target dataset. This culminates in the attainment of a predictive model tailored to the target dataset. In stage 1 of Figure 3, there are two encoders, one colour representing the input and output of one encoder. For example, in the first encoder, yellow represents the encoded result, white represents the input and output of the encoder (theoretically expected to be the same), and the middle green of the second encoder represents the encoded result. On the right are two decoders, with green representing the input and output of the first decoder, pink representing the input and output of the second decoder, and the final decoder in red.

## RESULTS

### Datasets and settings

Table 1 shows the datasets used in this paper. The human and mouse pancreatic single-cell data is sourced from studies conducted by Baron *et al.* [23] The mouse brain single-cell data with 11 cell types is sourced from studies conducted by Campbell *et al*. [24] The mouse brain single-cell data with seven cell types is sourced from studies conducted by Romanov *et al.* [25] To verify the performance of GDEC on across species，we used the human pancreatic dataset as the source data to pre-train GDEC, and then transferred the obtained model parameters to the mouse pancreatic dataset for transfer learning. Finally, we compared the predicted results with the labels provided. Similarly, we used two batches of brain datasets from mouse to validate the effectiveness of the model on across batches. The specific effects are detailed in sections Evaluations on cross species task and Evaluations on cross batch task. The detailed parameters of the model are shown in Table 2. The experiment was completed on a 2080Ti 11G graphics card with 32GB of memory.

**Table 1:** The details of dataset

Species	Tissue	Cell Num	Cell Types	Gene Num
Homo	Pancreatic islet	8569	14	789
Mouse	Pancreatic islet	1886	13	789
Mouse	Brain	21,086	11	801
Mouse	Brain	2881	7	801
Total	—	34,422	45	1590

**Table 2:** The parameters of GDEC

	Input	Output	Activation	Bias
Encoder	variable	500	ReLU	TRUE
	500	500	ReLU	TRUE
	500	2000	ReLU	TRUE
	2000	20	ReLU	TRUE
Assignment	20	label	None	None

### Evaluations on cross species task

We employed GDEC to transfer training data from human pancreatic data to mouse pancreatic data. Additionally, Seurat, Moana and scVI were used as comparative models to validate the effectiveness of our proposed model. We identified the intersection of cell types between the source and target datasets and selected the six cell types with the highest coverage across both datasets for our testing.

**Figure 4:**
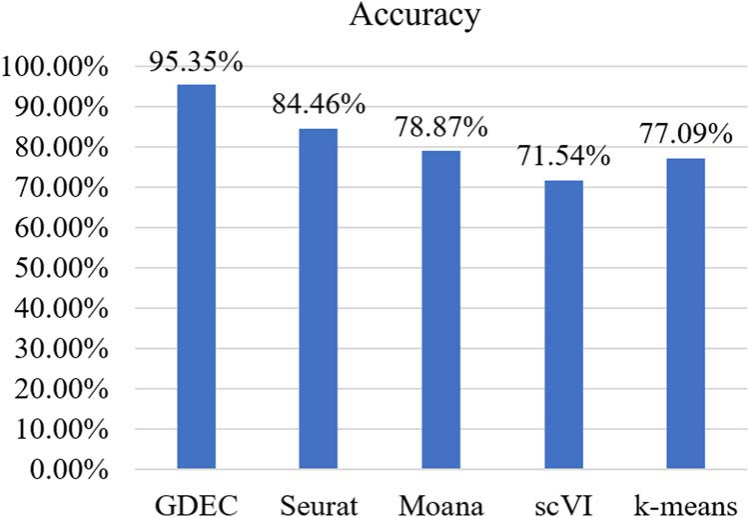
Cell clustering accuracy comparison on cross-species.

As depicted in Figure 4, the cell type identification results achieved by GDEC surpass those of other models. In order to elucidate the specific differences between traditional methods and our approach in actual predictions, we conducted further type matching and analysis on the GDEC and Moana methods.

**Figure 5:**
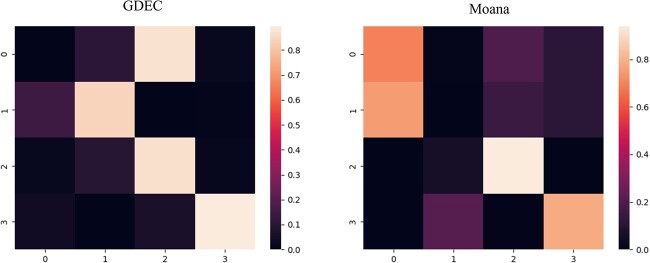
(**A**) Heatmap of cross-species clustering based on GDEC. (**B**) Heatmap of cross-species clustering based on Moana.

As shown in Figure 5, the color scale represents the proportion of the predicted number of samples with the current label to the total number of samples in all samples with the same label. The vertical axis label of the graph represents the true value, and the horizontal axis label represents the predicted value. Figure 5A showcases the predictive results of GDEC, where apart from the cell type labelled as 0, all other categories can be accurately identified. This is evident from the confusion matrix, highlighting that our proposed model attains remarkably high accuracy with minimal manual adjustments. Figure 5B presents the recognition outcomes of Moana, wherein it misclassifies three cell types within the label four cell type. Even with manual adjustments, it remains unable to accurately identify the correct cell types.

### Evaluations on cross batch task

**Figure 6:**
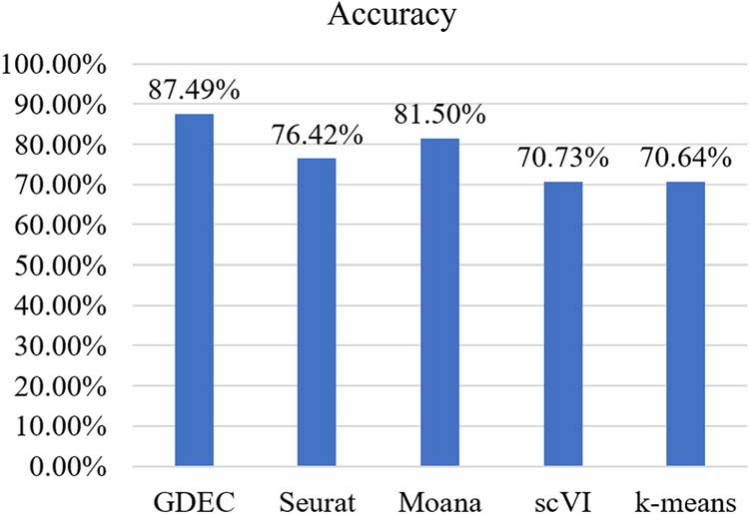
Cell clustering accuracy comparison on cross-batches.

In order to verify whether our model is applicable on different batches of data, we validated it on different batches of mouse brain datasets. As shown in Figure 6, the overall prediction accuracy of all models in the prediction of the brain is generally lower than that of the pancreas dataset, but GDEC still has the highest accuracy among all popular pipelines.

**Figure 7:**
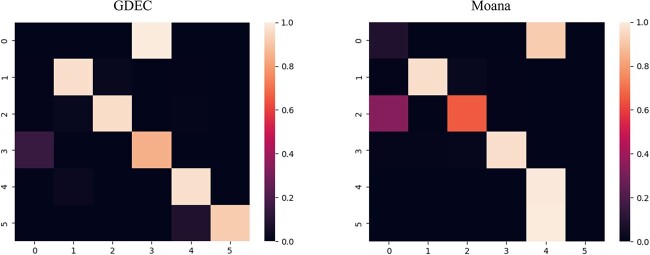
(**A**) Heatmap of cross-batch clustering based on GDEC. (**B**) Heatmap of cross-batch clustering based on Moana.

Since the accuracy of Moana is second only to GDEC, we compared the clustering results of the two (Figure 7). GDEC can get accurate results after simple human correction. But Moana’s predictions are hard to revise. For Moana’s prediction results, the prediction results of each label are chaotic and there is no clear distinction. For example, in all samples with a true value of 1, some are predicted to be 0, while others are predicted to be 2 and 3, indicating that the model did not distinguish this type of sample clearly. Even if secondary clustering is performed, significant different clusters cannot be obtained. Therefore, it is proved that our model can give more accurate predictions than other models, whether it is on the pancreas or another batch of brain data.

### Ablation study

**Figure 8:**
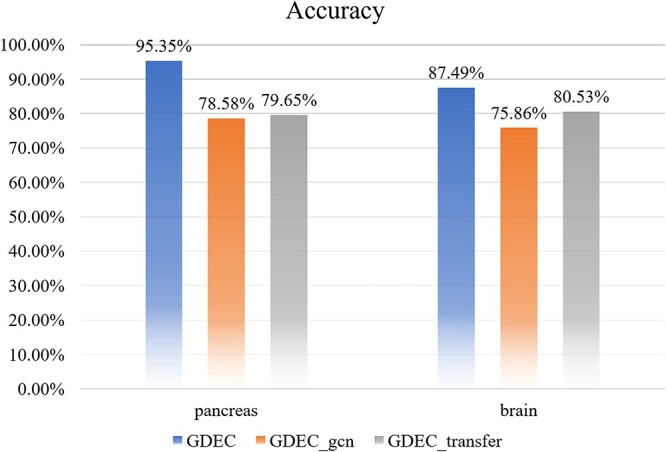
Cell clustering accuracy comparison on ablation study.

To establish the significance of each component within our model, we conducted ablation experiments. We removed the graph neural network feature extraction component from the model, referred to as GDEC_gcn, and omitted the transfer learning portion, making direct predictions on the target data, termed GDEC_transfer. As depicted in Figure 8, it is evident that GDEC outperforms others across all tasks.

Within the same dataset, GDEC_transfer outperforms GDEC_gcn, indicating that graph feature embeddings extract inter-gene relationships, thereby bolstering predictive outcomes. The absence of graph embedding features impedes achieving high accuracy, even when transferring knowledge from a high-quality dataset to the target dataset, underscoring the capability of graph embeddings to capture deeper latent relationships.

For the same model, GDEC_gcn exhibits superior performance on pancreatic data, implying that transfer learning excels at learning cross-species information, aligning with expectations as transfer learning is fundamentally designed to tackle similar problems. In GDEC_transfer, enhanced performance on the brain dataset is noted, signifying that graph embedding features more effectively extract intra-species gene relationships, with a slight decrement in cross-species relationships.

Through these comparisons, it is evident that graph embedding features excel in capturing inter-gene interaction relationships, while transfer learning adeptly addresses issues of dissimilar data distributions and similarity. The synergy of these two components yields excellent predictive results.

### Case on uterine fibroids

#### Comparison of GDEC and Seurat on uterine fibroids

We acquired single-cell sequencing data from the study conducted by Goad *et al.* [26], encompassing five normal uterine and five uterine leiomyoma tissues, resulting in a total of 100 664 cells. We used the uterine data from the study conducted by Huang *et al.* [27] as the source dataset for GDEC pre-training, encompassing six normal uterine，resulting in a total of 78 705 cells. By juxtaposing GDEC with the conventional processing pipeline Seurat, we elucidated the significance of accurate cell clustering for downstream biological knowledge extraction.

**Figure 9:**
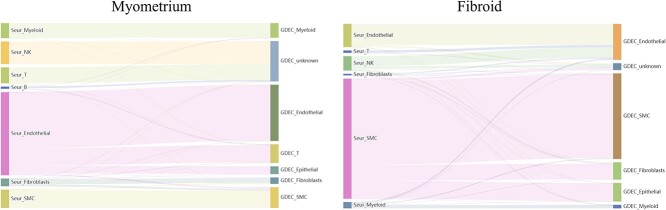
Proportion of different cell types in uterine fibroids and myometrium based on different methods.

In the normal tissue samples, myeloid, smooth muscle cells (SMC) and fibroblasts are distributed in a similar proportion across both methods (Figure 9). NK and B cells, absent in the training samples of GDEC’s source data, are clustered into distinct groups by the model and classified as unknown types. This highlights the ability of our model to reject classifying types instead of forcibly categorizing uncertain or unknown types as known. This capacity can be leveraged for guiding the classification of new cell types in future practical applications. Seurat’s identification of endothelial cells, further divided into epithelial and endothelial by GDEC, demonstrates that GDEC can achieve finer-grained cell classification.

Within the fibroid tissue, Seurat identifies SMC cells comprising nearly 70% while fibroblasts account for only 1%, contradicting factual observations (Figure 9). Conversely, GDEC identifies 10% of cells as fibroblasts, and all these cells are categorized as SMC by Seurat. This suggests Seurat’s limited ability to discern uncommon cell types. Alternatively, these cells may be in a fibrotic stage, exhibiting subtle changes that GDEC effectively captures. This bears significant clinical relevance for patient type diagnosis. Similarly, in fibroid tissue, Seurat fails to accurately distinguish SMC and epithelial cells, whereas GDEC can differentiate between the two.

Hence, it’s evident that GDEC provides a more detailed cell partitioning compared to Seurat. For uncommon cell types, GDEC offers more accurate predictions. With unknown cell types, GDEC can reject assigning them to a specific category instead of obligating a forced judgment like Seurat, offering significant value for exploring unknown cell types.

#### Functional changes of different cell types underlying uterine fibroids

We first used the Seurat method and GDEC method to classify cell types in normal and leiomyoma tissues, and then performed gene differential expression analysis based on cell types. By using KEGG to perform pathway analysis on the differential genes obtained, pathways with a *P*<0.05 were selected. Some new KEGG pathways were discovered by GDEC in fibroclasts cells and endothelial cells (not found by the Seurat method).

**Figure 10:**
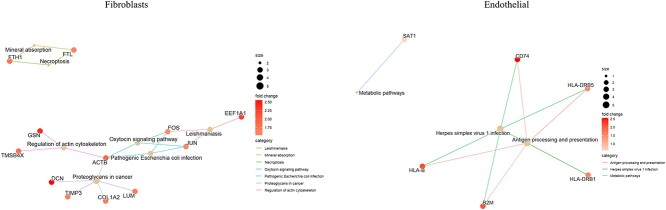
The KEGG pathway discovered through GDEC method, which are not discovered on Seurat.

New pathways such as proteoglycans in cancer and necroptosis have been discovered in fibroclasts cells, as shown in Figure 10. These pathways indicate the expression of genes related to cancer and necrosis, indicating that the patient’s tissue has undergone infection and carcinogenesis. However, no similar pathway was found in Seurat’s results, indicating that our model can detect changes in cell gene expression that traditional methods cannot detect. This is of great value for clinical diagnosis of tissue cancer and infection. Similarly, in endothelial cells, GDEC discovered the antigen processing and presentation, herpes simplex virus 1 infection, and metabolic pathways. These pathways indicate that the tissue has begun antigen processing and herpes virus infection, further speculating that the patient has already experienced infection. Through the above analysis, it is demonstrated that our model has the ability to discover new functions beyond traditional methods.

**Figure 11:**
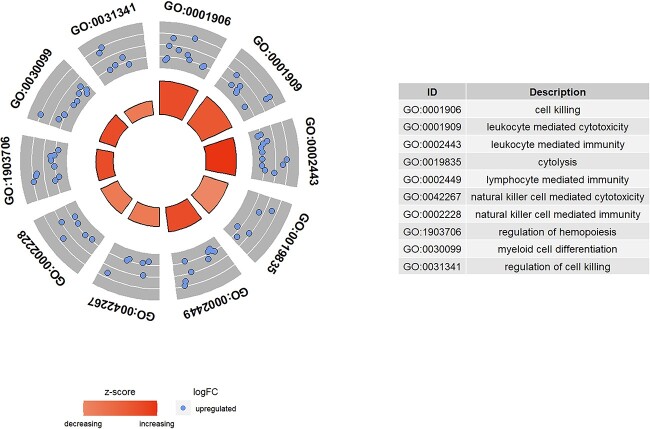
GO analysis on ‘unknown’ cell type.

**Figure 12:**
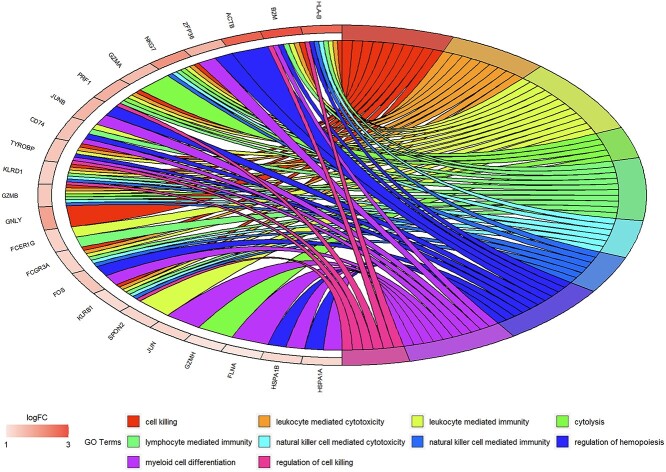
KEGG analysis on ‘unknown’ cell type.

We also conducted GO and KEGG enrichment analysis on cells classified as ‘unknown’ (Figures 11 and 12). Within the KEGG pathways, we observed pathways such as antigen processing and presentation, natural killer cell mediated cytotoxicity and apoptosis, all of which bear relevance to viral infections, immune responses and programmed cell death. Consequently, it can be deduced that the ‘unknown’ cell subset encompasses both virus-associated cells and immune effector cells. This concurs with our expectations, given the absence of immune cell annotations in the primary data, prompting the model to designate them as ‘unknown.’ Encouragingly, the ‘unknown’ category also unveils pathways implicated in cell apoptosis and rejection responses. These pathways likely denote infrequent yet distinctive functional cell subpopulations. Notably, identifying cells undergoing apoptosis poses challenges for conventional analytical tools, underscoring the model’s utility in facilitating novel explorations of prospective cell classifications.

## DISCUSSION

Clustering methods play a fundamental role in the analysis of scRNA-seq data by enabling the exploration of cellular heterogeneity, the discovery of novel cell types, and the illumination of biological processes. However, given that each cell captures transcripts from tens of thousands of genes and the sequencing coverage is often low, a substantial number of genes remain undetectable, leading to considerable noise in the data. Consequently, achieving accurate cell clustering presents a formidable challenge.

Most of the existing clustering techniques rely on conventional methods like Kmeans, tailored for cell clustering within the specific dataset. Yet, due to the noise and sparsity inherent in single-cell data, some approaches combine imputation methods with clustering techniques. Despite these efforts, limitations persist, and the full potential of numerous established gold standard datasets remains untapped.

In response, this paper introduces GDEC, a transfer learning-based clustering algorithm for single-cell data, which integrates GCN and DEC. GCN integrates gene interaction information to address sparse gene expression matrices, while DEC learns cell feature representations in a low-dimensional space to mitigate noise interference. Most notably, GDEC accomplishes cross-species and cross-batch clustering of single-cell data. Leveraging prior knowledge from target datasets, the model parameters are fine-tuned to facilitate transfer learning, harnessing the extensive repository of single-cell data for enhanced clustering precision. This method treats cell types that do not appear in the training set as unknown types, which is a methodological limitation. However, as the training set continues to expand, theoretically it can achieve recognition of all known cell types in humans. At the same time, this method can also provide hints for unknown cell types in humans, which is a significant improvement compared to other traditional methods.

To validate GDEC’s prowess, we first established a model using human pancreatic tissue scRNA-seq data and fine-tuned it using mouse pancreatic data, exemplifying cross-species migration learning. Experimental results highlight GDEC’s superior clustering accuracy compared to general toolkits, with the added benefit of easier correction of clustering errors through manual annotation. We extended this validation to a model built on mouse brain tissue data, followed by fine-tuning using another set of mouse brain data for cross-batch transfer learning. Once again, GDEC exhibited enhanced clustering accuracy compared to alternative toolkits. These experiments underscored GDEC’s effectiveness in cross-species and cross-batch clustering of single-cell data.

Illustrating GDEC’s utility in downstream analysis of single-cell data, we examined a uterine fibroid dataset. In contrast to clustering using Seurat packages, GDEC demonstrated heightened accuracy in distinguishing epithelial cells from endothelial cells. Additionally, for cell types absent from the training set, GDEC successfully clustered and researchers can identify them accurately by annotation tools. With GDEC’s aid, we unearthed a greater number of differentially expressed genes in specific cell types between uterine fibroids and normal tissues. This insight holds considerable value in elucidating the functional distinctions among cell types.

**Key Points**

- We fused graph convolutional networks (GCN) with deep embedding clustering (DEC) to construct graph-based deep embedding clustering (GDEC) to achieve cell clustering on single-cell RNA-sequencing data.
- Using transfer learning techniques to fine-tune the GDEC model, GDEC is enable to cluster scRNA-seq data cross different species and batches.
- Case on uterine fibroids shows that compared with traditional tool, GDEC can identify novel cell type and more accuracy cell type specific genes to discover novel function of cells.

## Data Availability

All data used in this paper can be downloaded from https://github.com/YuzhiSun/GDEC/tree/main.

## Notes

**YuMei Wang** is a deputy chief physician in Department of Gynecology and Shanghai Key Laboratory of Maternal and Fetal Medicine, Shanghai First Maternity and Infant Hospital, School of Medicine, Tong Ji University, Shanghai, China.

**Yuzhi Sun** is a PhD student in the Department of Computer Science at the Harbin Institute of Technology. His expertise is bioinformatics.

**Beiying Wang** is a deputy chief physician in Department of Gynecology and Shanghai Key Laboratory of Maternal and Fetal Medicine, Shanghai First Maternity and Infant Hospital, School of Medicine, Tong Ji University, Shanghai, China.

**Zhiping Wu** is an attending physician in Department of Gynecology and Shanghai Key Laboratory of Maternal and Fetal Medicine, Shanghai First Maternity and Infant Hospital, School of Medicine, Tong Ji University, Shanghai, China.

**XiaoYing He** is a deputy chief physician in Department of Gynecology and Shanghai Key Laboratory of Maternal and Fetal Medicine, Shanghai First Maternity and Infant Hospital, School of Medicine, Tong Ji University, Shanghai, China.

**Yuansong Zhao** worked as a Bioinformatician in the Beth Israel Deaconess Medical Center and is a research fellow in University of Texas Health Science Center at Houston.
